# Supplementary material for: Combining the HCT-CI, G8, and AML-Score for Fitness Evaluation of Elderly Patients with Acute Myeloid Leukemia: A Single Center Analysis
Source: Cancers (Basel). 2023 Feb 4;15(4):1002. doi: 10.3390/cancers15041002 (PMC9954486; doi:10.3390/cancers15041002)
Supplement: Supplementary file 1 [file cancers-15-01002-s001.zip › cancers-2085536-supplementary.pdf]

Supplementary figure.

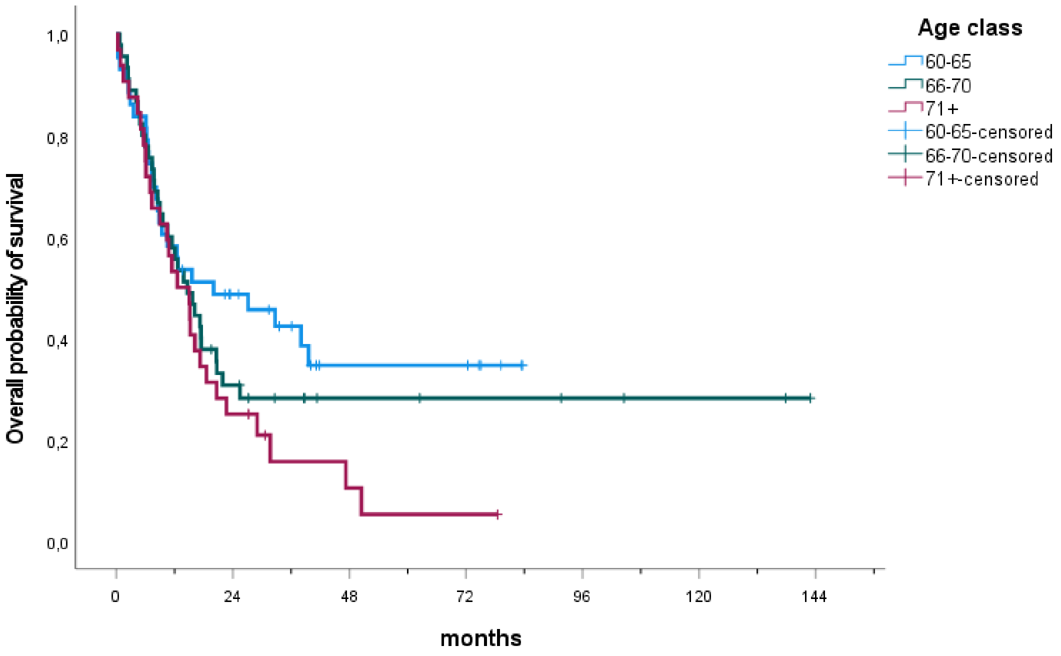

Supplementary Figure S1. Overall survival in different patient age groups. The 120 intensively treated patients were stratified according to their age. Their median OS decreased significantly with enhancing age. Patients between 60-65 years (n=43, 35.8%) had a longer median OS with 20.1 months (IQR: 6.7-not reached), compared to 66-70-year-old patients (n=45, 37.5%) with 14.6 months (IQR: 7.4-not reached) and ≥71-year-old patients (n=32, 26.7%) with a median OS of 12.6 months (IQR: 6.1-22.7), p=0.147.
